# Latest progress in low-intensity pulsed ultrasound for studying exosomes derived from stem/progenitor cells

**DOI:** 10.3389/fendo.2023.1286900

**Published:** 2023-11-28

**Authors:** Yi-fang He, Xia-li Wang, Shuang-ping Deng, Yan-li Wang, Qing-qing Huang, Shu Lin, Guo-rong Lyu

**Affiliations:** ^1^ Department of Ultrasound, The Second Affiliated Hospital of Fujian Medical University, Quanzhou, China; ^2^ Departments of Medical Imaging, Quanzhou Medical College, Quanzhou, China; ^3^ Centre of Neurological and Metabolic Research, The Second Affiliated Hospital of Fujian Medical University, Quanzhou, China; ^4^ Diabetes and Metabolism Division, Garvan Institute of Medical Research, Darlinghurst, Sydney, NSW, Australia

**Keywords:** stem cells, exosomes, low-intensity pulsed ultrasound, differentiation, proliferation, migration, homing

## Abstract

Stem cells have self-renewal, replication, and multidirectional differentiation potential, while progenitor cells are undifferentiated, pluripotent or specialized stem cells. Stem/progenitor cells secrete various factors, such as cytokines, exosomes, non-coding RNAs, and proteins, and have a wide range of applications in regenerative medicine. However, therapies based on stem cells and their secreted exosomes present limitations, such as insufficient source materials, mature differentiation, and low transplantation success rates, and methods addressing these problems are urgently required. Ultrasound is gaining increasing attention as an emerging technology. Low-intensity pulsed ultrasound (LIPUS) has mechanical, thermal, and cavitation effects and produces vibrational stimuli that can lead to a series of biochemical changes in organs, tissues, and cells, such as the release of extracellular bodies, cytokines, and other signals. These changes can alter the cellular microenvironment and affect biological behaviors, such as cell differentiation and proliferation. Here, we discuss the effects of LIPUS on the biological functions of stem/progenitor cells, exosomes, and non-coding RNAs, alterations involved in related pathways, various emerging applications, and future perspectives. We review the roles and mechanisms of LIPUS in stem/progenitor cells and exosomes with the aim of providing a deeper understanding of LIPUS and promoting research and development in this field.

## 1 Introduction

Stem cells, found in various tissues and organs, are undifferentiated cell populations originating from the early stages of embryonic development. They possess a limited capacity for self-renewal and demonstrate the ability to undergo multidirectional differentiation, giving rise to diverse specialized cell types. They can be classified into embryonic stem cells, induced pluripotent stem cells, and adult stem cells, with adult stem cells exhibiting a more limited differentiation potential. Stem cells undergo differentiation into progenitor cells, which possess a greater potential for differentiation but have limited self-renewal ability. Progenitor cells are characterized as undifferentiated cells with a higher proliferative capacity and the capability to differentiate into specific cell lineages (1). Mesenchymal stem cells (MSCs) are pluripotent tissue stem cells that can differentiate into a variety of mesodermal tissue types and are present as a type of adult stem cell in various mature tissues *in vivo*, such as the bone marrow, adipose, umbilical cord, and teeth, and they represent the most widely studied class of stem cells (2). Stem cells can exert paracrine effects by secreting factors, such as cytokines and inflammatory factors, and immunomodulatory effects by interacting with immune cells. Their low immunogenicity makes them immune to rejection at the time of transplantation (3). In some cases, inflammatory stimuli cause MSCs to suppress or enhance the ability of the immune response to localize to the site of inflammation, thus exerting an anti-inflammatory effect (4).

Exosomes are extracellular vesicles with a bilayer membrane structure, diameter of 40–100 nm, and density of 1.13–1.19 g/mL. They contain proteins, mRNAs, miRNAs, and DNA. Exosomes are present in nearly all cells and body fluids and are generally obtained from various types of cells, such as stem cells. Exosomes are important for intercellular communication, and they are involved in the integration of cells in physiological and pathological states by delivering biomolecules, thereby causing a series of biochemical changes in receptor molecules. They function in immune regulation (5), reproduction (6), tumor angiogenesis (7), cell differentiation and regeneration (8), apoptosis (9), and inflammatory responses (10). MiRNAs are endogenous non-coding single-stranded RNAs of 21–23 nucleotides that bind directly to the 3′-untranslated regions (3′ UTRs) of target mRNAs and regulate post-transcriptional gene expression negatively or positively (11). Most cells can secrete miRNAs, as can MSC-derived exosomes (MSC-EXOs). MSC-EXOs help restore dynamic cellular homeostasis by delivering proteins, lipids, and other information and affect the proliferation, differentiation, migration, and other behaviors of stem cells through various mechanisms that lead to altered signaling pathways (12, 13).

All of these different types of stem/progenitor cells have promising clinical applications in regenerative medicine (14). MSC-EXOs have the same properties as stem cells but lower immunogenicity, tumorigenicity, and infectivity, and they express MSC surface molecules CD90, CD44, and CD73; moreover, MSCs and extracellular vesicles have similar miRNA expression profiles (15, 16). Therefore, MSC-EXOs are widely studied and applied in regenerative medicine and various diseases (17). Studies have elucidated the biological roles of miRNA-mediated MSC-EXO effects in various tissues. A recent review concluded that MSC-EXO miRNAs have a dual role in the cancer microenvironment, where they significantly inhibit tumorigenesis and transfer between donor and recipient cells, leading to cancer chemoresistance, thus providing new ideas for identifying strategies for overcoming cancer drug resistance (18).

Endometrium-derived MSC-EXOs with high levels of miRNAs are involved in the regulation of macrophage polarization, T-cell activation, and inflammatory cytokine transcription by the immune system (19). MSC-EXO miR-140-5p regulates the mTOR pathway by targeting IGF1R (20), thereby inhibiting the osteogenic differentiation of MSCs. In addition, exosomes and their secreted miRNAs can serve as carriers for drug delivery and have been applied in various disease treatments (21, 22). However, stem cell and exosome therapies still have limitations, such as insufficient sources, mature differentiation, low transplantation success, and insufficient target organ homing, which limit their clinical application (23, 24). In addition, the treatment mechanism is not fully understood in many cases and the amount of treatment required for different diseases has not been standardized (25). To address these issues, methods to improve homing rates are increasingly being explored.

**Figure 1:**
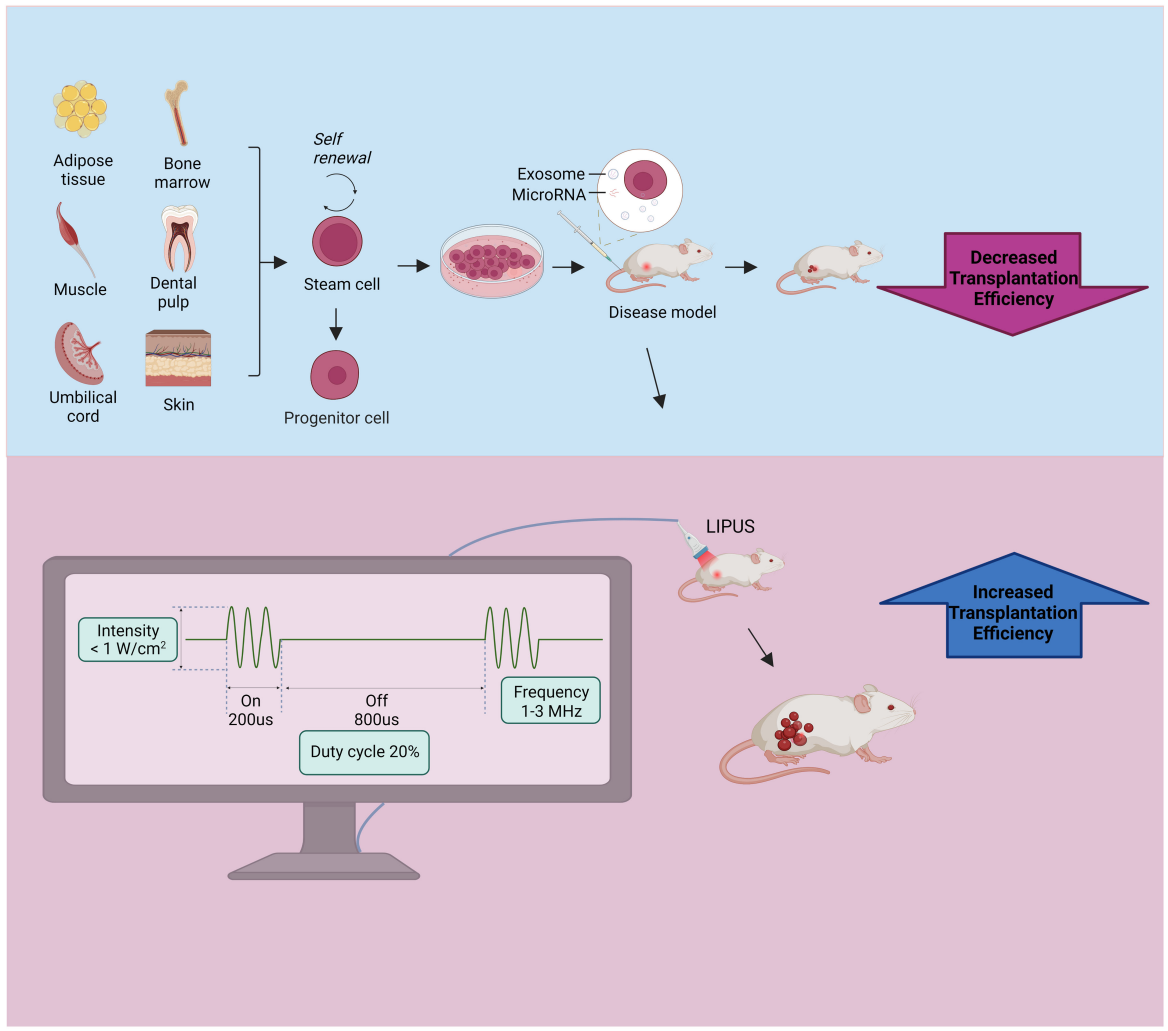
Stem cell and exosome injections are used to treat disease models, and further exposure to LIPUS can increase transplantation efficiency. The stem cell source is shown in the upper left, the lower left shows the common parameters of LIPUS, which features an intensity of less than 1 W/cm^2^, a frequency of 1-3 MHz, and a duty cycle of 20%. LIPUS, low-intensity pulsed ultrasound.

Low-intensity pulsed ultrasound (LIPUS) is a special type of ultrasound output in the form of pulsed waves with frequencies of 1 to 3 MHz and intensities less than 1 w/cm^2^, and it can be used as a non-invasive physical stimulus for therapeutic applications (26). LIPUS has a low thermal effect because of its low intensity and pulsed output mode; thus, it presents limited thermal effects while delivering acoustic energy to the target tissue. Its mechanical and cavitation effects provide low-intensity mechanical vibrational stimuli that interact with cells and trigger numerous intracellular changes, such as the release of cytokines and signaling molecules. These changes can alter the cellular microenvironment and affect biological behaviors, such as cell proliferation, differentiation, and migration, leading to tissue repair and regeneration (27). LIPUS is a promising treatment modality that was initially used for skeletal muscle disorders and has been shown to promote fracture healing, cartilage repair in osteoarthritis (OA), and tendon ligament injury recovery (28, 29). A recent review summarized the role and mechanism of LIPUS in the repair of peripheral nerve injury (PNI), and as a non-invasive stimulation method, LIPUS is expected to be a successful alternative treatment for PNI with great advantages and application prospects (30). In recent years, studies have found that LIPUS has important effects on stem/progenitor cells and exosomesis emerging as an important tool for enhancing stem cell therapy (**Figure 1**).

Herein, we review the research progress in the use of LIPUS for stem/progenitor cell exosomes from three aspects: (1) the effects of LIPUS on the biological functions of stem/progenitor cells and exosomes; (2) the signal transduction pathways in stem/progenitor cells and exosomes affected by LIPUS; and (3) the prospects of clinical applications of LIPUS combined with stem/progenitor cells and exosomes. This review aims to provide a deeper understanding of LIPUS and promote research and development in this field.

## 2 Effects of LIPUS on the biological functions of stem/progenitor cells

**Figure 2:**
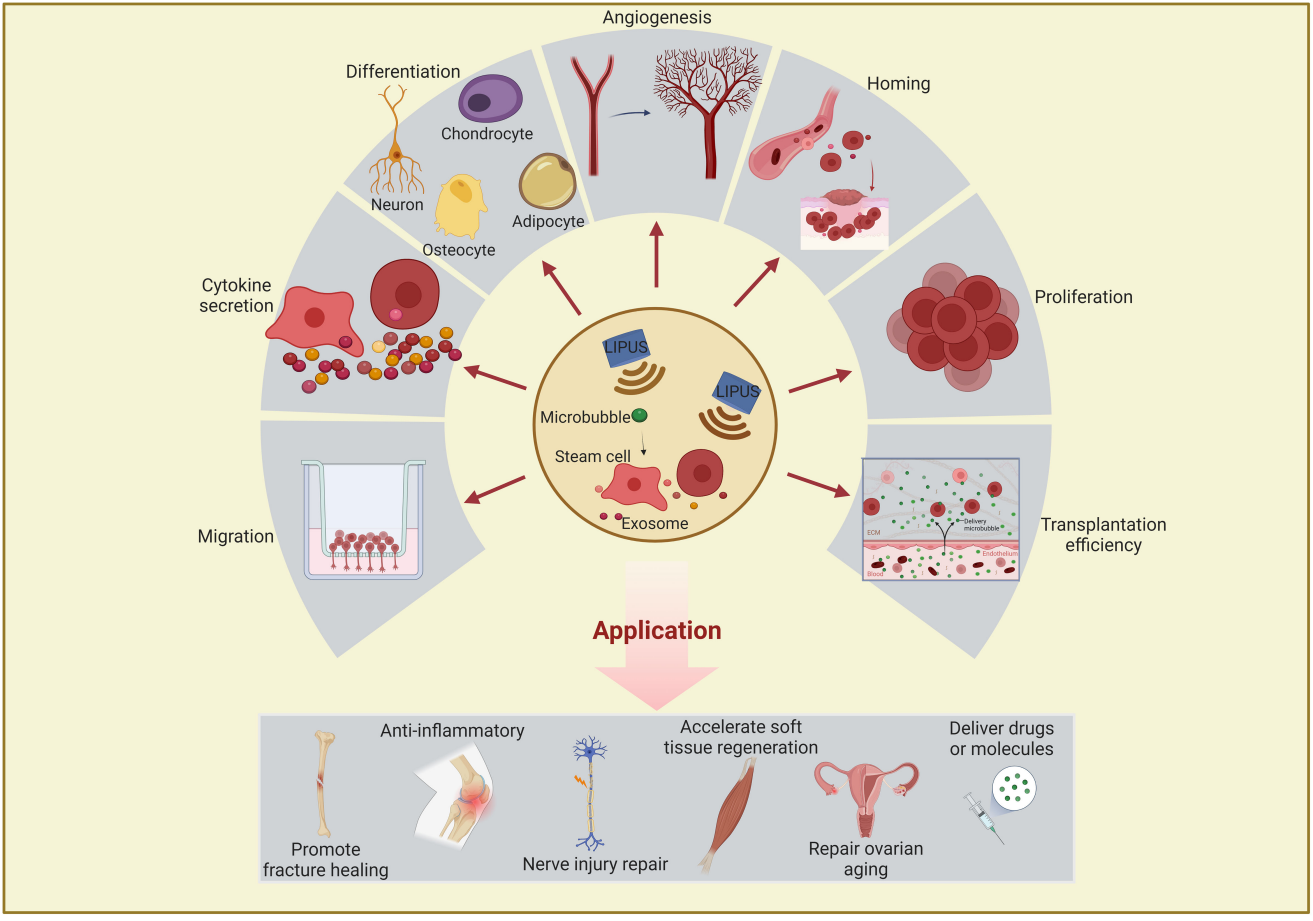
Schematic representation of the effects and applications of LIPUS or combined with microbubble on stem/progenitor cells and exosomes. LIPUS affects stem/progenitor cells and exosomes in the following areas: differentiation, proliferation, migration, homing, transplantation efficiency, cytokine secretion, and angiogenesis regulation. These functions are closely related to therapeutic applications in regenerative medicine and often used to promote fracture healing, anti-inflammation, nerve injury repair, soft tissue regeneration, and drug delivery. LIPUS, low-intensity pulsed ultrasound.

Studies have revealed that LIPUS can affect stem/progenitor cell differentiation, migration, and proliferation and exosome functions through mechanical stimulation (31–33). These biological functions are described in detail below and summarized in **Figure 2** and **Tables 1**–**4**.

**Table 1:** The effect of LIPUS on the differentiation of stem cells.

Cell type	LIPUS parameters				Injection method	effects on stem cells	Signaling pathway	Disease	Year of publication	References
	Intensity (mW/cm^2^)	Frequency(MHz)	Duty ratio (%)	Time(mins)						
BMSCs	–	3	20	20	–	Chondrogenic differentiation	Integrin-mTOR signaling pathway	–	2017	(34)
BMSCs	–	3	20	20	–	Chondrogenic differentiation	–	–	2019	(35)
BMSCs	100	1	10	30	–	Proliferation, adhesion, osteogenic differentiation	–	–	2018	(36)
BMSCs	1000/1500	–	–	0.5/1	–	Cell viability, differentiation	Wnt/β-catenin signaling pathway	–	2018	(37)
BMSCs	30	0.6	20	20	–	Differentiation, proliferation	–	–	2019	(38)
BMSCs	100	1	20	120	–	Cell viability, osteogenic differentiation	RhoA/ROCK signaling pathway	osteoradionecrosis of the jaw	2020	(39)
BMSCs	1000	1	20	1	–	Differentiation, Repulsiveness	–	–	2020	(40)
ADSCs	100	1	20	8	–	Osteogenic differentiation	–	–	2012	(41)
ADSCs	100	1	20	10	–	Osteogenic differentiation	–	–	2013	(42)
ADSCs	100	1	20	20	–	Lipogenic differentiation	–	–	2013	(43)
ADSCs	20/30	2	20	30	–	Osteogenic differentiation	–	bone defect	2018	(44)
PDLSCs	90	1.5	20	10/20/30	–	Osteogenic differentiation	–	–	2014	(45)
PDLSCs	90	1.5	–	20	–	Osteogenic differentiation	–	periodontitis	2017	(46)
PDLSCs	–	–	–	–	–	Osteogenic differentiation	–	–	2021	(47)
PDLSCs	90	1.5	20	15/30	–	Osteogenic differentiation	NF-κB signaling pathway	–	2022	(48)
PDLSCs	50	1.5	20	20	–	Endothelial differentiation, angiogenesis	–	-	2022	(49)
PDLSCs	90	1.5	–	30	–	Osteogenic differentiation	–	periodontitis	2020	(50)
HGPCs	30	1.5	–	–	–	Neural differentiation	–	–	2014	(51)
C2C12 myoblast	–	–	–	–	–	Proliferation, differentiation	–	–	2017	(52)
NSCs	69.3	1	–	–	–	Proliferation, differentiation	Notch signaling pathway	–	2020	(53)
iMSC	40	1.5	50	10	–	Osteogenic differentiation	–	–	2022	(54)
iPSCs-NCSCs	300/500	1	20	–	–	Proliferation, neural differentiation	FAK-ERK1/2 signaling pathway	peripheral nerve defect	2019	(55)
NSPCs	533	–	20	–	–	Attachment, differentiation	–	–	2019	(56)
HCC CSC	–	1.132	20	–	–	Differentiation, reduced invasiveness	–	–	2019	(57)
NSCs	–	–	20	5-15	–	Neuronal differentiation	ERK1/2 signaling pathway	–	2023	(58)

LIPUS, Low-intensity pulsed ultrasound; mins, minutes; BMSCs, Bone marrow-derived mesenchymal stem cells; ADSCs, Adipose-derived mesenchymal stem cells; PDLSCs, Periodontal ligament stem cells; HGPCs, Human gingival progenitor cells; NSCs, Neural stem cells; iPSCs-NCSCs, Pluripotent stem cell-derived neural crest stem cells; NSPCs, Neural stem/progenitor cells; HCC CSCs, hepatocellular carcinoma cancer stem cells.

**Table 2:** The effect of LIPUS on the other biological functions of stem/progenitor cells.

Cell type	LIPUS parameters				Injection method	effects on stem cells	Signaling pathway	Disease	Year of publication	References
	Intensity (mW/cm^2^)	Frequency(MHz)	Duty ratio (%)	Time(mins)						
BMSCs	50/60	1.5	20	5	–	Proliferation	P13K/AKt signaling pathways	–	2019	(33)
BMSCs	100	1	10	10	–	Proliferation, adhesion, osteogenic differentiation	–	–	2018	(36)
BMSCs	30	0.25	20	20	tail vein	Migration	FAK-ERK1/2 signalling pathway	femoral defects	2019	(59)
BMSCs	30	1.5	–	–	tail vein	Migration, homing	–	alveolar bone defect	2022	(60)
BMSCs	–	1.02	–	7.3	–	Cell viability	–	–	2019	(61)
BMSCs	50	1	–	3	injured epicenter(invasive)	Cell viability, migration, nerve growth factor expression	–	contused spinal cord	2019	(62)
BMSCs	25	1.11	20	20	–	Influence of immediate early gene (IEGs) expression	MAPK/ERK signaling pathway	–	2020	(63)
MSCs	100	1.5	20	–	–	Proliferation, chondrodifferentiation	–	–	2018	(64)
MSCs	30	1.5	20	20	intracardiac injection (confirmed by ultrasound)	Migration, recruitment	SDF-1/CXCR4 signaling pathway	fracture	2014	(32)
MSCs	50	3	20	20	intra-articular injection	Migration	–	osteoarthritis	2021	(65)
MSCs	–	1	5	–	tail vein	Homing	–	–	2020	(66)
MSCs	60	1.5	–	10	–	Proliferation	–	distraction osteogenesis	2022	(67)
ADSCs	30	1.5	20	5	–	Proliferation	–	–	2020	(68)
ADSCs	300	5	–	10	–	Proliferation	–	–	2022	(69)
ADSCs	70/210	0.5	–	1	–	Cell viability, apoptosis	–	–	2019	(70)
ADSCs	15.5	1	20	–	–	Angiogenesis	–	–	2019	(71)
ADSCs	20	1	20	10	–	The myelination capacity of Schwann cells (SC)	–	peripheral nerve injuries	2016	(72)
ADSCs	30	1.5	–	20	fibrin glue during the operation	Bone healing	–	bone tendon healing	2019	(73)
ADSCs	200	1.7	20	5	intracavernosal injection	Proliferation, secretion of cytokines	Piezo-ERK-VEGF signaling pathway	erectile dysfunction	2022	(74)
hAD-MSCs	–	0.25	20	30	–	Proliferation	ERK1/2 and PI3K-Akt signalling pathways	–	2017	(75)
hAD-MSCs	30	0.25	20	30	tail vein	Growth factor secretion, improved ovarian function	–	primary ovarian insufficiency	2017	(76)
PDLSCs	90	1.5	20	30	–	Migration	SDF1/CXCR4 signaling pathway	–	2018	(77)
PDLSCs	30	–	–	10	–	Anabolic effects	–	–	2012	(78)
DPSCs 、PDLSCs、BMSC	250/750	1	–	5/20	–	Proliferation	MAPK signaling pathway	–	2016	(79)
DPSCs 、PDLSCs	250/750	1	–	–	–	Proliferation	Piezo-mediated regulation of ERK1/2 MAPK signaling pathway	–	2017	(80)
hUC-MSCs	–	–	20	–	–	Proliferative, secretory activity	–	–	2022	(81)
SSCs	–	–	–	–	–	Proliferation	–	–	2017	(82)
C2C12 mesenchymal precursors	44.5	3.6	27.8	5	–	Proliferation	–	–	2018	(83)
ESCs	10~30	1.5	20	–	–	Proliferation, osteogenic differentiation, mineralized tissue formation	–	–	2022	(84)
GBMCSCs	500	1	20	1	–	Cell viability	PI3Ka/AKT/mTOR signaling pathway	–	2022	(85)
GSCs	300	1.5	20	20/5	–	Increased sensitivity of GSC to temozolomide	–	–	2022	(86)

LIPUS, Low-intensity pulsed ultrasound; mins, minutes; BMSCs, Bone marrow-derived mesenchymal stem cells; MSCs, Mesenchymal stem cells; ADSCs, Adipose-derived mesenchymal stem cells; hAD-MSCs, Human amniotic mesenchymal stem cells; PDLSCs, Periodontal ligament stem cells; DPSCs, Dental pulp stem cells; hUC-MSCs, Human umbilical cord mesenchymal stem cells; SSCs, Spermatogonial stem cells; ESCs, Embryonic stem cells; GBMCSCs, Glioblastoma cancer stem cells; GSCs, Glioma stem cells.

**Table 3:** The effect of LIPUS combined with microbubbles on the biological function of stem/progenitor cells.

Cell type	LIPUS parameters				Injection method	effects on stem cells	Signaling pathway	Disease	Year of publication	References
	Intensity (mW/cm^2^)	Frequency(MHz)	Duty ratio (%)	Time (mins)						
BMSCs	2000	1	–	–	–	Migration, homing	–	–	2018	(87)
BMSCs	1500	1	20	10	tail vein	Homing	–	acute liver injury	2018	(88)
BMSCs	2000	1	50	2	tail vein	Cellular Viability	–	acute myocardial infarction	2020	(89)
BMSCs	2000	1	–	2	tail vein	Homing	–	acute myocardial infarction	2021	(90)
BMSCs	600	1	10	–	–	Migration, homing	–	acute kidney injury	2016	(91)
BMSCs	23	–	10	5	tail vein	Homing	–	chronic bacterial prostatitis	2016	(92)
MSCs	–	–	–	–	–	Migration	–	–	2020	(93)
MSCs	30	1.5	20	1-5	tail vein	Proliferation, chondrogenic differentiation	–	–	2016	(94)
MSCs	30	1.5	20	3	–	Proliferation, osteogenic differentiation	–	–	2019	(95)
MSCs	600	1	10	0.5	tail vein	Migration, homing	SDF-1/CXCR4 signaling pathway	myocardial infarction	2015	(96)
NSCs	–	18	–	30	–	Neural differentiation	Piezo1-Ca2+ -BMP2/Smad signaling pathway	–	2022	(48)

LIPUS, Low-intensity pulsed ultrasound; mins, minutes; BMSCs, Bone marrow-derived mesenchymal stem cells; MSCs, Mesenchymal stem cells; NSCs, Neural stem cells.

**Table 4:** The effect of LIPUS on the biological function of stem/progenitor cells or exosomes.

Cell type	LIPUS parameters				Injection method	effects	Signaling pathway	miRNA	Disease	Year of publication	References
	Intensity (mW/cm^2^)	Frequency (MHz)	Duty ratio (%)	Time (mins)							
BMSC-EXO	30	1.5	20	20	intraarticular injection	Cartilage regeneration	NF-κB signaling pathway	–	osteoarthritis	2021	(97)
MSC-EXO	50	3	20	20	intraarticular injection	Promotion of exosome release	–	–	osteoarthritis	2022	(98)
BMDCs-EXO	30	1.5	–	–	–	Enhancement of exocrine secretion	NF-κB signaling pathway	miR-16, miR-21	–	2019	(99)
hMSCs	30	1.5	–	–	–	Signal transduction, osteogenesis	HIF-1a Signaling pathway	miR-31-5p	–	2019	(100)
OCSCs	1000	1	10	1	–	miRNA delivery	–	miR-let7b	–	2018	(101)
hPDLC	90	1.5	–	–	–	Osteogenic differentiation	–	miR-182	–	2019	(102)
BMSCs	300	1	20	15	dripped on the wound	Generation of EV	MAPK signaling pathway	miR-328-5p, miR-487b-3p	allogeneic skin transplantation model	2023	(103)

LIPUS, Low-intensity pulsed ultrasound; mins, minutes; BMSCs, Bone marrow-derived mesenchymal stem cells; EXO, Exosome; MSCs, Mesenchymal stem cells; BMDCs, Bone marrow dendritic cells; OCSCs, Ovarian cancer stem cells; hPDLCs, Human primary periodontal cells.

### 2.1 Effects of LIPUS on the differentiation of stem/progenitor cells

#### 2.1.1 Bone marrow-derived MSCs (BMSCs)

BMSCs are multipotent stem cells. An et al. (36) stimulated rat BMSCs with 100 mW/cm^2^ LIPUS and found increases in mineralized nodules in the extracellular matrix and the expression levels of osteogenic-related genes encoding the bone-bridging protein osteopontin (OPN), osteocalcin (OCN), bone morphogenetic protein-2 (BMP-2), alkaline phosphatase (ALP), Runt-related transcription factor 2 (Runx2), and type 1 collagen, suggesting that LIPUS promotes the osteogenic differentiation of BMSCs on titanium surfaces. Zhang et al. (39) found that LIPUS reverses the effects of radiation on the osteogenic differentiation of rat mandible-derived BMSCs. Yao et al. (104) reported a cyclic arginine-glycine-aspartate-modified nanobubble that could actively target BMSCs via integrin receptors and was used in combination with LIPUS to further enhance the osteogenic differentiation and bone formation of BMSCs induced by LIPUS. He et al. (38) used LIPUS to irradiate BMSCs with a frequency of 0.6 MHz, a duty cycle of 20%, and an intensity of 30 mW/cm^2^ and showed that LIPUS treatment promoted the differentiation efficiency and rate of BMSCs. Song et al. (40) and Li et al. (37) treated BMSCs with hepatocyte growth factor and found that LIPUS significantly upregulated the levels of the liver markers alpha-fetoprotein, cytokeratin 18, albumin, and glycogen in the BMSCs, indicating that LIPUS-induced BMSC differentiation towards hepatocytes. Autophagy is involved in regulating BMSC differentiation into chondrocytes. Xia et al. (34) and Wang et al. (35) found that LIPUS inhibited autophagy in BMSCs by regulating the integrin pathway and autophagy to promote the chondrogenic differentiation of BMSCs.

In summary, these studies suggest that LIPUS can regulate the differentiation of BMSCs towards osteogenic, hepatocytic, and chondrogenic lineages. The effects of LIPUS appear to be mediated through various cellular signaling pathways, gene expression, and autophagy. However, further research is needed to elucidate the mechanisms and potential applications of LIPUS-induced BMSC differentiation.

#### 2.1.2 Adipose-derived stem cells (ADSCs)

ADSCs from subcutaneous fat are more readily available than BMSCs and have a greater capacity for proliferation and differentiation (105). Yue et al. (42) and Zhang et al. (44) isolated mouse and human ADSCs *in vitro* and stimulated the cells *in vitro* with a certain intensity of LIPUS. These authors performed protein blotting analysis of osteogenic-related genes and found that LIPUS promoted mineralized nodule formation and upregulated the osteogenic-related genes *Runx2*, *OCN*, *ALP*, and *OPN*, bone sialo protein, and heat shock protein (HSP) 70, HSP90, BMP-2, and BMP proteins. These results suggest that LIPUS stimulation can enhance the osteogenic differentiation of ADSCs by upregulating the expression of HSP70 and HSP90 and activating the BMP signaling pathway. Fu et al. (43) cultured mouse ADSCs in medium containing adipogenic reagents and stimulated them with 30 mW/cm^2^ LIPUS, and an analysis of adipogenic genes and proteins revealed that LIPUS upregulated the adipocyte lipogenic factors peroxisome proliferator-activated receptor γ and adiponectin, suggesting that LIPUS promotes the lipogenic differentiation of ADSCs.

#### 2.1.3 Other stem/progenitor cells

Kusuyama et al. (46) extracted periodontal ligament stem cells (PDLSCs) from three healthy third molars and used LIPUS to intervene with BMP9 to induce PDLSC differentiation, which was found to be effective in promoting osteogenic differentiation under inflammatory conditions. LIPUS with an intensity of 90 mW/cm^2^ and a frequency of 1.5 MHz promoted the osteogenic differentiation of PDLSCSs both *in vitro* and *in vivo* (47, 50). Hu et al. (49) demonstrated that 50 mW/cm^2^ LIPUS promoted the endothelial differentiation and angiogenesis of PDLSCs under inflammatory or non-inflammatory conditions. Lee et al. (56) demonstrated for the first time that intense dual-frequency LIPUS exposure promotes neural stem/progenitor cell differentiation and growth factor utilization more than single-frequency LIPUS owing to the cavitation effect. Recently, they found that dual-frequency ultrasound regulates calcium channels via the downstream extracellular signal-regulated kinase 1/2 (ERK1/2) pathway, thereby promoting the differentiation of functional neural stem/progenitor cell neurons and the secretion of brain-derived neurotrophic factor (BDNF) (58). Furthermore, they found that dual-frequency ultrasound affects cancer stem cells (CSCs) by inducing CSC differentiation and reducing drug resistance and invasiveness; thus, it represents an alternative therapeutic option for treating human tumors (57). Wu et al. (53) found that LIPUS at an intensity of 69.3 mW/cm^2^ and a frequency of 1 MHz not only reduced astrocyte differentiation but also stimulated neuronal differentiation *in vitro* by modulating the Notch signaling pathway. Xia et al. (55) applied a rat sciatic nerve injury model and demonstrated that LIPUS may regulate the proliferation of induced pluripotent stem cell-derived neural crest stem cells (iPSC-NCSCs) via the focal adhesion kinase (FAK)-ERK1/2 signaling pathway and neural differentiation, suggesting that LIPUS may be a useful alternative approach in the field of neural regeneration. In 2022, Hua et al. (54) demonstrated for the first time that LIPUS promoted osteogenic differentiation in iPSC-derived MSCs, and the optimal parameters were an intensity of 40 mW/cm^2^, a frequency of 1.5 MHz, and a duty cycle of 50%.

### 2.2 Effects of LIPUS on the proliferation of stem/progenitor cells

#### 2.2.1 MSCs

Huang et al. (68) found that LIPUS at 30 mW/cm^2^ upregulated the gene expression of proliferation-related proteins cyclin D1, c-Myc, and stromal cell-derived factor (SDF)-1α in ADSCs, thereby promoting their proliferation and extending their duration in an undifferentiated state. The proliferative effect of LIPUS on ADSCs has also been demonstrated by Min et al. (69). The effect of LIPUS on the proliferation of PDLSCs has been demonstrated in several studies. For example, Gao et al. (79, 80) found that LIPUS promoted the proliferation of MSCs of different origins by activating different MAPK pathways in an intensity- and cell-specific-dependent manner and revealed that these odontogenic MSCs all had Piezo membrane ion channels sensitive to mechanical stimulation, suggesting that the proliferation of LIPUS-stimulated odontogenic MSCs may involve piezoelectric regulation of ERK1/2 signaling. Han et al. (67) treated rat MSCs with LIPUS at a power of 60 mW/cm^2^ and a frequency of 1.5 MHz in combination with BMP-2 and assessed relevant proliferation and osteogenic indicators. They found that the combination more strongly promoted cell proliferation, and they validated the effect of the combination treatment in a rabbit distraction osteogenesis model. Ling et al. (75) isolated MSCs from human placental amnion and stimulated them with LIPUS at 30 mw/cm^2^ and found that LIPUS promoted the transition of MSCs from the G0/G1 phase to the S and G2/M phases and the proliferation of cells. Recently, Ren et al. (81) isolated MSCs from human umbilical cord and found that LIPUS was effective in stimulating cell proliferation and secretory activity *in vitro*; moreover, LIPUS-treated MSCs effectively reduced thyroid cell apoptosis and excessive autoimmune antibody accumulation, and improved thyroid function in a rat experimental autoimmune thyroiditis model.

#### 2.2.2 Other stem/progenitor cells

Salgarella et al. (52) exposed C2C12 myogenic cells to different regimens of LIPUS and found that stimulation at 1 W/cm^2^ and 3 MHz maximized cell proliferation. Puts et al. (83) found that stimulation of mouse C2C12 mesenchymal precursors with LIPUS at 44.5 mW/cm^2^ and 3.6 MHz promoted cell proliferation. Detection of Yes-associated protein (YAP), which acts as a mechanosensor in C2C12 cell fate, revealed increased levels of YAP in the nucleus, whereas silencing of YAP expression eliminated the beneficial effects of LIPUS, suggesting that LIPUS enhances cell proliferation potential by regulating YAP function, which is essential for tissue regeneration processes. The installation of LIPUS modules in a bioreactor for large-scale production of 3D tissue structures based on embryonic stem cells promoted stem cell proliferation, osteogenic mineralized tissue formation, and cavity filling in a rabbit cranial defect model (84). Moghadam et al. (82) found that LIPUS with a mechanical index of 0.4 was effective in increasing the proliferation rate of spermatogonial stem cells within 7 days of culture while a higher mechanical index was detrimental to the cells, suggesting that LIPUS may be an option for the treatment of male oligospermia.

### 2.3 Effects of LIPUS on the migration of stem/progenitor cells

The effect of LIPUS on stem/progenitor cell migration has been confirmed by many studies. Wang et al. (77) isolated PDLSCs from premolar teeth and found that LIPUS at an intensity of 90 mW/cm^2^ promoted PDLSC migration based on wound healing and Transwell assays. Subsequently, they injected BMSCs via the tail vein, irradiated the defective areas in rats directly with 30 mW/cm^2^ LIPUS, and assessed alveolar bone regeneration using micro-computed tomography and found that LIPUS promoted periodontal alveolar bone regeneration based on the BMSCs, suggesting that LIPUS provided treatment by improving the homing and migration of BMSCs (60). Chen et al. (59) applied LIPUS at 30 mW/cm^2^ to stimulate BMSCs *in vitro* and then injected the BMSCs into rats with femoral defects followed by LIPUS intervention. Their study revealed that LIPUS promoted BMSC migration *in vitro* and *in vivo*, which was possibly associated with FAK-ERK1/2 pathway activation. In 2021, Xia et al. (65) also demonstrated that LIPUS promoted MSC migration using *in vivo* and *in vitro* experiments and found that LIPUS at 50 mW/cm^2^ activated autophagy, which could be inhibited by the use of autophagy inhibitors. In a rat knee OA model, the combined application of LIPUS and BMSCs significantly promoted OA cartilage repair, and this effect was attenuated by autophagy inhibitors, suggesting that LIPUS promotes BMSC migration and OA cartilage repair through autophagy regulation.

### 2.4 Effects of LIPUS on other biological functions of stem/progenitor cells

In addition to its effects on stem/progenitor cell differentiation, proliferation, and migration, LIPUS affects other biological functions, such as cell viability, cytokine secretion, and tissue homing. In recent years, the optimization of MSC homing and their secretion of therapeutic molecules have been explored, with LIPUS representing an emerging technology (106). One study revealed that LIPUS increased the survival rate of rat BMSCs by 19.57%, with a further 5.36% increase after the optimization of LIPUS parameters to 6.92 V, 1.02 MHz, and 7.3 min (61). LIPUS-stimulated BMSCs showed increased BDNF expression and cell viability *in vitro*, and better functional recovery was found in a rat spinal cord injury model treated with LIPUS combined with BMSCs, suggesting a possible application of LIPUS in the treatment of spinal cord injury (62). Song et al. (86) stimulated glioma stem cells (GSCs) with LIPUS at 300 mW/cm^2^ and 1.5 MHz, and showed that LIPUS resulted in diminished expression of GSC biomarkers and promoted GSC escape from G0 quiescence. They further performed experiments on nude mice and confirmed that LIPUS enhanced the sensitivity of GSCs to temozolomide both *in vivo* and *in vitro*. To promote angiogenic tissue regeneration, Kang et al. (71) prepared a scaffold composed of collagen and acetyl hyaluronate for coculture of human ADSCs and umbilical vein endothelial cells (HUVECs). Under LIPUS stimulation at 15.5 mW/cm^2^ *in vitro*, 1.85-fold and 1.5-fold increases in collagen and cell growth were observed, respectively. These effects were validated in a rat angiogenesis model, indicating that LIPUS can promote the angiogenic capacity and therapeutic potential of scaffold-based cocultured ADSCs/HUVECs.

### 2.5 Effects of LIPUS combined with microbubbles on stem/progenitor cells

Ultrasound targeted microbubble destruction (UTMD) is a promising technique for non-invasive targeted therapy. Microbubbles are an ultrasound imaging contrast agent that are injected to trigger microbubble cavitation combined with low frequency ultrasound to increase biofilm permeability in the vicinity of the microbubble, thereby facilitating the delivery of biomolecules to cells through blood vessels and achieving targeted therapeutic effects. In recent years, numerous studies have confirmed the effects of UTMD on stem/progenitor cells, particularly in improving their homing ability and transplantation efficiency, effectively addressing the limitations of inadequate homing of seed cell target organs and low transplantation success. Osborn et al. (95) used 30 mW/cm^2^ LIPUS in combination with microbubbles to intervene in hMSCs and found that it significantly enhanced the proliferation of cells in 3D-printed scaffolds. The effects of LIPUS combined with microbubbles on the homing, migration, and survival of stem/progenitor cells have also been demonstrated in many studies. In animal models of chronic bacterial prostatitis, acute liver injury, and acute myocardial infarction, LIPUS combined with microbubbles promoted the homing of BMSCs to damaged tissues, thus enhancing treatment efficacy (88–90, 92). Cui et al. (93) injected microbubbles and MSCs into rats with brain defects and subjected them to LIPUS transcranial irradiation of the cerebral ischemic zone. They found that the MSCs attached to and crossed the blood-brain barrier of the cerebral vasculature to reach the brain parenchyma to improve neurobehavioral functions. As an excellent delivery vehicle, microbubbles not only protect biomolecules from endogenous clearance during transport but also allow for controlled release in target organs through inertial cavitation and have promising applications.

## 3 Effects of LIPUS on exosomes

The effects of LIPUS on MSC-EXOs have also been demonstrated. MiRNAs are closely related to various physiological and pathological processes in humans, and the effect of LIPUS on miRNAs has been confirmed in numerous studies.

### 3.1 Effects of LIPUS on stem/progenitor cell-derived exosomes

Xia et al. (98) recently isolated MSCs from rat bone, cocultured them with OA chondrocytes, and intervened with an inhibitor of exosome release. They then stimulated the cells with LIPUS and detected exosome release and other autophagy markers by transmission electron microscopy. The results suggested that LIPUS promotes exosome release from MSCs via autophagy activation. In a rat knee OA model, they observed that LIPUS significantly enhanced the positive effect of MSCs on OA cartilage, which was significantly blocked by the exosome release inhibitor GW4869. *In vitro* and *in vivo* experiments showed that LIPUS enhances the therapeutic effect of MSCs in OA cartilage repair by a mechanism related to the promotion of autophagy-mediated exosome release. The study revealed that LIPUS did not impact the shape and size of exosomes, but it increased the release of exosomes from MSCs for the appropriate duration of stimulation. However, the authors did not investigate how LIPUS enhances autophagy in MSCs to increase the number of exosomes within them, and how LIPUS affects interactions and communication between MSCs and OA chondrocytes through the exosome release pathway to further promote cartilage repair. Future research should delve into these areas to provide valuable insights and guidance for utilizing LIPUS in cartilage repair applications.

Li et al. (103) found that LIPUS stimulation resulted in a 3.66-fold increase in exosome release from BMSCs, and they demonstrated the enhanced anti-inflammatory effects of LIPUS-stimulated BMSC-EXOs both *in vivo* and *in vitro*. BMSC RNA-Seq analysis revealed that LIPUS enhanced the cytoskeletal activity of BMSC, leading to increased secretion of bioactive factors, including exosomes. Small RNA-Seq analysis of LIPUS-stimulated BMSC-EXOs identified differentially expressed miRNAs mainly involved in cellular activities. LIPUS-stimulated BMSC up-/down-regulated genes may inhibit inflammation through different mechanisms, whereas miR-328-5p and miR-4876-3p up-regulated in LIPUS-stimulated exosomes target the MAPK signaling pathway to regulate inflammatory responses. However, these mechanisms require further experimental validation. Investigating the interactions between LIPUS-stimulated BMSC-derived exosomes and target cells such as immune cells or damaged tissues is essential to understand their anti-inflammatory effects. This study may involve exploring the uptake mechanisms and downstream signaling pathways activated by LIPUS-stimulated exosomes in recipient cells.

Liao et al. (97) stimulated BMSC-EXOs with LIPUS at 30 mW/cm2 and 1.5 MHz and found that it promotes the anti-inflammatory properties of BMSC-EXOs in OA. This enhancement effect further improved the synthesis and proliferation of extracellular matrix in chondrocytes, thereby promoting the regeneration of articular cartilage. This study suggests that LIPUS may exert its effects by inhibiting the nuclear factor-κB (NF-κB) pathway activated by IL-1β. However, further studies are needed to explore the specific targets, doses, and intrinsic mechanisms of LIPUS-mediated BMSC-EXOs. In addition, this study was conducted in 6-week-old pups. Young rats have a greater capacity for regeneration and their skeletal system is not yet fully developed. Therefore, it may be worth considering whether it is necessary to conduct experiments on adult animals. Li et al. (99) treated bone marrow dendritic cells (BMDCs) with LIPUS using the same parameters and incubated HUVECs with their secreted exosomes and found that the exosomes from the LIPUS-treated BMDCs were enriched in miR-16 and miR-21 and phagocytosed by the HUVECs, prevented tumor necrosis factor (TNF)-α-induced HUVEC activation, and downregulated the expression of cell adhesion molecules. These changes prevented TNFα-induced activation of the NF-κB signaling pathway and ultimately suppressed endothelial inflammation. However, this study was limited to cellular level investigations and further studies are needed to explore the conditions required for *in vivo* experiments. Moreover, investigating the effects of LIPUS stimulation on exosome uptake by HUVECs and the subsequent modulation of cell adhesion molecule expression to inhibit endothelial cell activation would deepen our understanding of the potential of LIPUS-stimulated exosomes for the treatment of relevant diseases.

### 3.2 Effect of LIPUS on miRNAs

MiRNAs are involved in various physiological and pathological processes in humans, and the effect of LIPUS on miRNAs has been confirmed in numerous studies. Costa et al. (100) first investigated the role of LIPUS as a promoter of miRNA expression, identified miR-31-5p as a LIPUS-inducible miRNA through bioinformatics analysis, and confirmed these findings by gain- and loss-of-function experiments. *In vitro* studies showed that miR-31-5p is capable of inducing hypoxia and cytoskeletal responses. Conversely, combined treatment with LIPUS and miR-31-5p inhibitors eliminated the hypoxic response, suggesting that miR-31-5p may be a LIPUS-mechanosensitive miRNA. Thus, LIPUS may be a novel therapeutic option to promote or eliminate the hypoxic response and cytoskeletal organization of hMSCs during bone regeneration. Chen et al. (102) stimulated periodontal ligament cells with LIPUS at 90 mW/cm^2^ and 1.5 MHz and found that it promoted the accumulation of cellular forkhead box O1 (FOXO1) protein and upregulated the expression of the osteogenic-related genes *ALP* and *Runx2*. MiR-182 downregulated FOXO1 through post-transcriptional regulation. Overexpression of miR-182 reversed LIPUS-induced FOXO1 expression and osteogenic differentiation, whereas LIPUS repressed miR-182 expression, which played a crucial role in promoting osteogenic differentiation, thus providing insights for applying LIPUS to periodontal bone defects.

Yang et al. (101) used the UTMD method to transfect miR-let7b into ovarian CSCs. Flow cytometry revealed that UTMD increased the transfection rate of miR-let-7b and the late apoptosis rate of CD133+ ovarian CSCs and decreased surface marker expression of CD133-expressing stem cells. These findings suggest that UTMD-mediated miRNA delivery may be a promising platform for the treatment of CSCs.

MiRNAs in exosomes are often mediators of the ultimate biological effects during LIPUS-promoted secretion. One study revealed that LIPUS remarkably increased the secretory and anti-inflammatory effects of BMSC-EXOs. RNA-sequencing analysis revealed significant upregulation of miR-328-5p and miR-487b-3p, thereby inhibiting the MAPK signaling pathway (103). This result suggests an important role for these two miRNAs in the mechanism underlying the enhancement of the anti-inflammatory effect, which provides insights into inflammation-related disease treatment.

## 4 Mechanisms involved in the biological effects of LIPUS on stem/progenitor cells and exosomes

**Figure 3:**
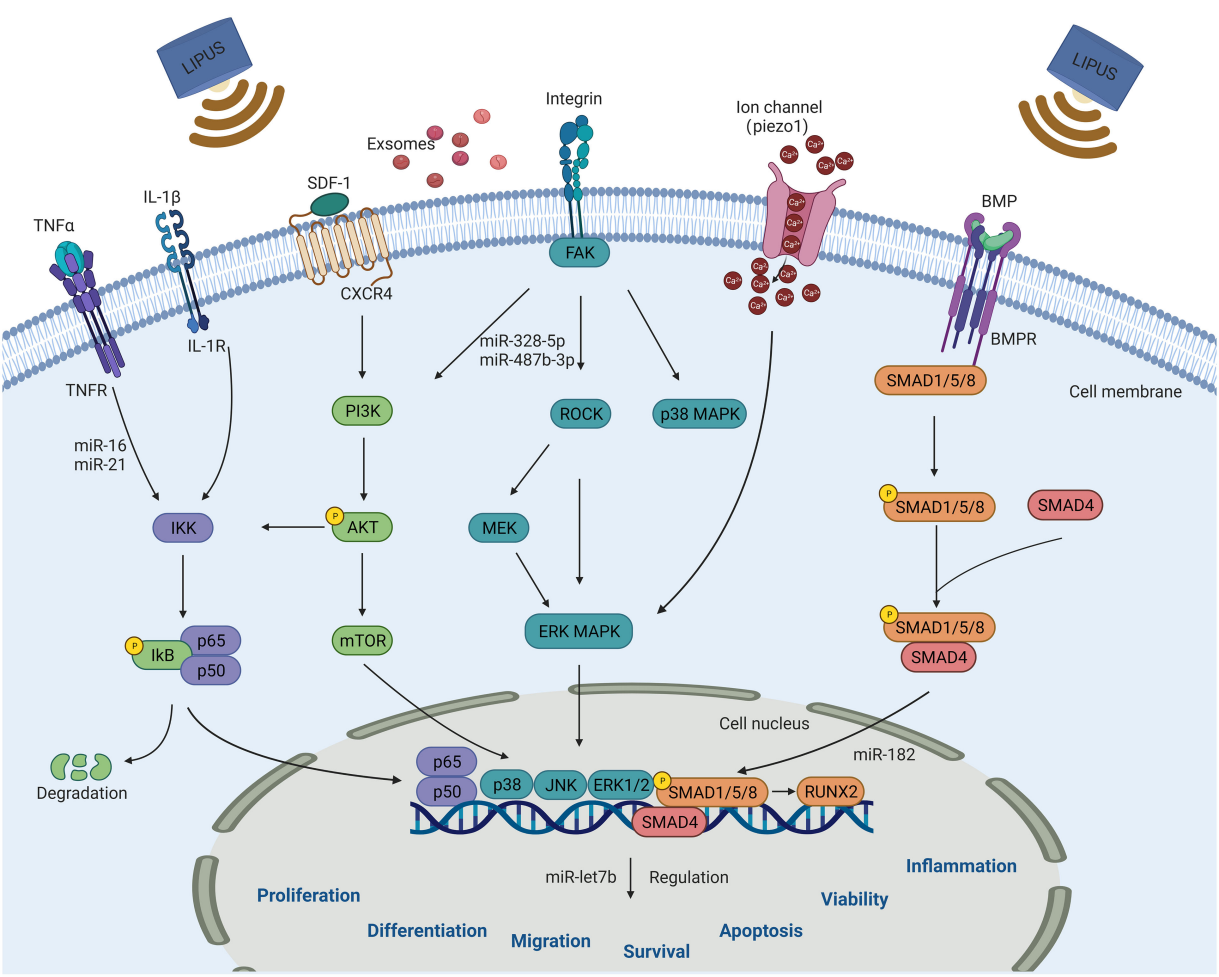
Schematic diagram of signaling pathways that can be activated by LIPUS in steam/progenitor cells for regulating cell biological functions, including proliferation, differentiation, migration, survival, apoptosis, vitality and inflammation. These pathways include the NF-κB, PI3K/AKT, MAPK, BMP/Smad, SDF-1/CXCR4, and Piezo-mediated signaling pathways. LIPUS, low-intensity pulsed ultrasound.

The mechanisms by which LIPUS affects the biological functions of stem/progenitor cells and exosomes are complex and not fully understood, although they are likely related to its mechanical and cavitation effects. The mechanical vibrations produced by LIPUS allow for micromechanical interactions with cells, triggering a series of intracellular biochemical events, such as the release of cytokines and signaling molecules, which in turn alter the cellular microenvironment and lead to changes in signaling pathways that affect a range of cellular biological behaviors (27). Currently known signaling pathways regulated by LIPUS include the MAPK, ERK, phosphoinositide 3-kinases (PI3K)/protein kinase B, stromal cell-derived factor 1 (SDF-1)/C-X-C chemokine receptor type 4 (CXCR4), and NF-κB signaling pathways, as shown in **Figure 3**.

### 4.1 MAPK signaling pathway

The MAPK signaling pathway plays an important role in regulating physiopathological processes, such as cell growth, differentiation, stress, and inflammatory responses, and its three important downstream components are ERK, Jun amino-terminal kinase (JNK), and p38. Tabuchi et al. (63) exposed mouse BMSCs to 25 mW/cm^2^ LIPUS and found a significant increase in immediate early gene expression, whereas the use of MAPK/ERK inhibitors prevented LIPUS-induced expression of FOS and EGR1. Wang et al. (70) investigated the changes in cell viability and apoptosis of ADSCs after different doses of LIPUS irradiation and found that a high dose of LIPUS (210 mW/cm^2^) promoted ADSC apoptosis while a low dose (70 mW/cm^2^) increased ADSC viability. The use of p38 MAPK activity inhibitors rescued the apoptotic effect of high doses of LIPUS, suggesting that p38 MAPK plays a key role in the effect of LIPUS on ADSCs. The effect of LIPUS on stem/progenitor cell proliferation and its mechanisms have been demonstrated in several studies. Using different pathway blockers, Gao et al. (79, 80) found that LIPUS promoted the proliferation of different types of MSCs via different MAPK signaling pathways and activated JNK MAPK signaling in BMSCs, ERK1/2 signaling in dental pulp stem cells (DPSCs), and JNK and p38 signaling in PDLSCs. They later demonstrated that LIPUS-stimulated proliferation of DPSCs involved Piezo-mediated regulation of ERK1/2 MAPK signaling. Xia et al. (55) found that LIPUS promoted the proliferation and neural differentiation of iPSC-NCSCs in rats with sciatic nerve injury, probably via the FAK-ERK1/2 signaling pathway.

Lee et al. (58) recently found that dual-frequency ultrasound could precisely regulate calcium channels via the downstream ERK1/2 signaling pathway, thereby promoting neuronal differentiation and BDNF secretion in NSCs. Another study revealed that the combination of LIPUS and ADSCs applied to rats with diabetes mellitus and erectile dysfunction significantly improved the treatment efficacy and promoted vascular endothelial growth factor secretion by ADSCs via the Piezo-ERK pathway (74).

### 4.2 PI3K/AKT signaling pathway

LIPUS has been shown to promote the proliferation of amniotic MSCs. Ling et al. (75) demonstrated that this proliferative effect induced by LIPUS was significantly reduced when ERK1/2 and PI3K inhibitors were used, suggesting the involvement of the ERK1/2 and PI3K/AKT signaling pathways. Specifically, PI3K, which is generally activated by upstream signaling, triggers a signal upon activation that not only alters the AKT protein structure but also activates downstream substrate alterations. These changes ultimately regulate crucial cellular processes such as proliferation, differentiation, migration, and other functions. These findings were further supported by a study conducted by Xie et al. (33), confirming the important role of the ERK1/2 and PI3K/AKT signaling pathways in LIPUS-mediated cell proliferation. In addition, the PI3K/AKT signaling pathway is involved in the effect of LIPUS on the survival of CSCs. The PI3K/AKT/mTOR pathway is often active in glioblastoma, and suppressing the cell survival of glioblastoma CSCs plays a key role in tumor therapy. Tutak et al. (85) found that high doses of anticancer drugs combined with LIPUS inhibited mTOR expression and reduced cell viability, and they concluded that the combined action of the drugs and LIPUS was achieved via the PI3K/AKT/mTOR signaling pathway.

### 4.3 SDF-1/CXCR4 signaling pathway

In regenerative medicine, stem cells are recruited to areas of injury to perform their biological functions, and cell migration, homing, and chemotaxis play an important role in this process. The chemokine SDF-1 and its receptor CXCR4 are key factors in regulating stem cell migration. Wei et al. (32) demonstrated that LIPUS upregulated the expression of SDF-1 and CXCR4 in rat MSCs and promoted the migration of MSCs to fracture sites, which could be attenuated by pathway blockers. Li et al. (96) found that the combined application of LIPUS and microbubbles increased the expression of SDF-1 in ischemic myocardium and upregulated CXCR4 expression on the surface of MSCs *in vitro* and *in vivo*, suggesting that their combined application may promote MSC homing to repair ischemic myocardium via the SDF-1/CXCR4 signaling pathway. Ling et al. (107) recently found that LIPUS promoted SDF-1-induced migration of human amniotic MSCs via the SDF-1/CXCR4 axis. In the ovaries of rats with chemotherapy-induced premature ovarian insufficiency (POI), SDF-1 levels were significantly elevated and LIPUS-treated MSC homing to the ovaries increased, thereby reducing ovarian damage and improving ovarian function. Conversely, CXCR4 antagonists reduced the number of MSCs homing to POI ovaries, reducing their effectiveness in POI treatment.

### 4.4 NF-κB signaling pathway

The NF-κB signaling pathway plays an important role in immune regulation, inflammatory and stress responses, and apoptosis. Liao et al. (97) investigated the mechanism by which LIPUS stimulates BMSC-EXOs to promote cartilage regeneration in OA and found that LIPUS-mediated BMSC-EXOs promoted chondrocyte proliferation and extracellular matrix synthesis and inhibited interleukin-1β-induced activation of the NF-κB signaling pathway, thereby suppressing inflammation. However, this study was conducted in 6-week-old mice, which are known to possess enhanced regenerative capacities and have immature skeletal systems. Therefore, this study limits the direct applicability of the results to adult humans or skeletally mature individuals. Another study identified the role for LIPUS in immunomodulation and osteogenesis in hPDLSCs and showed that LIPUS enhanced the immunomodulation and osteogenic differentiation of hPDLSCs by inhibiting the NF-κB signaling pathway in a dose-dependent manner (48).

### 4.5 Other signaling pathways

In addition to the above pathways, various other pathways have been investigated in relation to the biological functions of LIPUS in stem/progenitor cells and exosomes, such as the Piezo1-Ca^2+^-BMP2/Smad (108) and Notch (53) signaling pathways. Although these signaling pathways have been elucidated, the mechanisms underlying their interaction and the most important pathways for disease treatment remain unclear. In the future, *in vivo* implantation models can be used to validate their involvement and investigate their mechanisms of action. For example, *in vivo* models could be utilized to assess the effects of LIPUS on activation of stem/progenitor cell signaling pathways and subsequent functional outcomes. In addition, investigating the crosstalk between these pathways and other known pathways involved in disease processes would help to elucidate their interconnected roles.

LIPUS stimulation modulates signaling and cellular processes, thereby influencing the synthesis and release of exosomes and the composition, quantity, and cargo of bioactive molecules. This enhances the role of exosomes in intercellular communication and disease therapy. However, further validation of its mechanisms and a comprehensive understanding of its role are needed as research in this area is still in its early stages. Although existing studies suggest that LIPUS has therapeutic potential in exosome production, more research is needed to fully elucidate its detailed mechanisms and clinical applications.

By gaining a comprehensive understanding of these signaling pathways and their interplay, researchers can identify key targets for therapeutic intervention and develop more effective strategies for the treatment of various diseases.

## 5 Prospects and limitations of LIPUS for clinical applications

LIPUS is an attractive modality for treatment because it is non-invasive and has many advantages, including no risk of infection or tissue damage and no known adverse effects; thus, it is increasingly being explored in basic research and clinical applications. LIPUS has achieved promising results in the treatment of disorders of the skeletal muscular system. Clinical studies have revealed that LIPUS contributes to accelerated bone healing (109), joint function restoration in OA (110), rapid motor recovery in patients with lumbar spondylolisthesis (111), and spinal fusion (112). In addition, studies in preclinical animal models have shown that LIPUS can promote recovery and improve functional outcomes in PNI (30) and holds great potential in the prevention and recovery of traumatic encephalopathy (113–115). Du et al. (116) constructed a first-order characteristic model based on an apparent diffusion coefficient map to evaluate the therapeutic effect of LIPUS on acute craniocerebral injury, and it may be valuable in predicting the therapeutic effect of LIPUS in clinical practice.

The effects of LIPUS on stem cell proliferation, differentiation, migration and the associated signaling pathways have been previously discussed. When combined with stem cells, it can alleviate the problems of insufficient stem cell sources, maturity differentiation, low transplantation success, and insufficient target organ homing. Encouragingly, a large body of evidence suggests that LIPUS combined with stem cell and exosome therapies has positive effects in skeletal muscle system disorders and neurological injury and promising potential in suppressing inflammation and ovarian ageing recovery. In addition, LIPUS combined with microbubbles is often used for drug delivery or gene transfer, where small molecules such as miRNAs are loaded on microbubbles and act on the target organ under the effect of LIPUS cavitation. Compared to conventional methods, LIPUS cavitation can achieve superior therapeutic effects, such as increased sensitivity to chemotherapeutic agents and reduced resistance of CSCs.

Studies have shown satisfactory synergistic effects of LIPUS on stem/progenitor cells, although such methods are still in their infancy. A study registered in the Chinese Clinical Trials Registry in 2019 represented the first clinical study to comprehensively investigate the safety and efficacy of LIPUS for stem cell therapy in OA patients, thus representing the first breakthrough in translating combined LIPUS and stem cell therapy from animal models to clinical practice applications (117). Based on present studies, we recognize that the impact of different LIPUS parameters varies and that the transition from basic to clinical requires additional parameter optimization. Hopefully, a standard report of parameters will be established to help researchers explore this field in depth. Extensive further study is required for basic science research on LIPUS to move towards large-scale clinical dissemination and application.

## 6 Conclusion and future perspectives

New technologies to enhance the efficacy of stem/progenitor cells are of increasing interest to researchers. The effects of LIPUS on stem/progenitor cells and exosomes and the signaling pathways related to the mechanism have been confirmed by numerous studies. The combination of LIPUS with stem/progenitor cells and exosomes may improve the efficacy of treatment for certain diseases, although it is still in its preliminary stages. This review focused on basic experiments, most of which were cellular experiments. For the translation from basic research to clinical application, the number of animal studies should be increased in the future, with a shift from small animal models to large animal models and then to clinical trials. It is worth noting that in most animal experiments involving the use of stem cells and their extracellular vesicles for therapeutic purposes, tail vein injection or invasive local injections are commonly employed. However, we advocate for non-invasive local injections guided by ultrasound, which offer higher accuracy, safety, and reduced trauma. In addition, different LIPUS parameters have different effects on stem/progenitor cells and exosomes; therefore, identify appropriate therapeutic parameters must be identified. Hopefully, a standardized parameter reporting system will be established to promote more in-depth exploration. Efforts must be made to develop a convenient and inexpensive method to address the bottleneck of low graft success rates in the field of stem cell transplantation. In the future, we hope that more research results will support the positive therapeutic effect of LIPUS combined with stem/progenitor cells and exosomes and the popularization of this method in clinical practice. LIPUS is expected to be an important auxiliary tool to improve the therapeutic effect of stem/progenitor cells and exosomes.

## Author contributions

Y-FH: Writing – original draft, Writing – review & editing. X-LW: Writing – original draft. S-PD: Writing – review & editing. Y-LW: Writing – original draft. Q-QH: Writing – original draft. SL: Writing – review & editing. G-RL: Writing – review & editing.
